# Mucosal antibody responses to vaccines targeting SIV protease cleavage sites or full-length Gag and Env proteins in Mauritian cynomolgus macaques

**DOI:** 10.1371/journal.pone.0202997

**Published:** 2018-08-28

**Authors:** Hongzhao Li, Yan Hai, So-Yon Lim, Nikki Toledo, Jose Crecente-Campo, Dane Schalk, Lin Li, Robert W. Omange, Tamara G. Dacoba, Lewis R. Liu, Mohammad Abul Kashem, Yanmin Wan, Binhua Liang, Qingsheng Li, Eva Rakasz, Nancy Schultz-Darken, Maria J. Alonso, Francis A. Plummer, James B. Whitney, Ma Luo

**Affiliations:** 1 Department of Medical Microbiology and Infectious Diseases, University of Manitoba, Winnipeg, MB, Canada; 2 Center for Virology and Vaccine Research, Beth Israel Deaconess Medical Center, Harvard Medical School, Boston, MA, United States of America; 3 Center for Research in Molecular Medicine and Chronic Diseases (CIMUS), Campus Vida, Universidade de Santiago de Compostela, Santiago de Compostela, Spain; 4 Scientific Protocol Implementation Unit, Wisconsin National Primate Research Center, Madison, WI, United States of America; 5 National Microbiology Laboratory, Public Health Agency of Canada, Winnipeg, MB, Canada; 6 Nebraska Center for Virology, School of Biological Sciences, University of Nebraska-Lincoln, Lincoln, NE, United States of America; 7 Department of Biochemistry and Medical Genetics, University of Manitoba, Winnipeg, MB, Canada; 8 Immunology Services Unit, Wisconsin National Primate Research Center, Madison, WI, United States of America; 9 Ragon Institute of MGH, MIT, and Harvard, Cambridge, MA, United States of America; Emory University School of Medicine, UNITED STATES

## Abstract

HIV mutates rapidly and infects CD4^+^ T cells, especially when they are activated. A vaccine targeting conserved, essential viral elements while limiting CD4^+^ T cell activation could be effective. Learning from natural immunity observed in a group of highly HIV-1 exposed seronegative Kenyan female sex workers, we are testing a novel candidate HIV vaccine targeting the 12 viral protease cleavage sites (PCSs) (the PCS vaccine), in comparison with a vaccine targeting full-length Gag and Env (the Gag/Env vaccine) in a Mauritian cynomolgus macaque/SIV model. In this study we evaluated these vaccines for induction of mucosal antibodies to SIV immunogens at the female genital tract. Bio-Plex and Western blot analyses of cervicovaginal lavage samples showed that both the PCS and Gag/Env vaccines can elicit mucosal IgG antibody responses to SIV immunogens. Significantly higher increase of anti-PCS antibodies was induced by the PCS vaccine than by the Gag/Env vaccine (p<0.0001). The effect of the mucosal antibody responses in protection from repeated low dose pathogenic SIVmac251 challenges is being evaluated.

## Introduction

Development of an effective vaccine to human immunodeficiency virus type 1 (HIV) has proven to be a daunting task. Of the six HIV vaccine trials, the RV144 trial was the only one that demonstrated a modest efficacy (31.2%) [1, 2]. HIV primarily targets activated CD4^+^ T cells—a major arm of the immune system [1], apart from its capacity to mutate to evade immune recognition and generate extensive sequence variability [3–5]. Thus, a HIV vaccine generating immune response to conserved, functionally essential viral elements [6], and in the meantime limiting the generation of viral target cells [7, 8], could be more effective [1, 9–18].

The HIV protease cleaves Gag, Gag-Pol and Nef precursor proteins at twelve protease cleavage sites (PCSs) during viral maturation [18, 19]. The process is highly specific, temporally regulated and essential for generating infectious viral particles [20–25]. Even subtle disturbance can be sufficient to interrupt this delicately balanced process and drive it toward a non-productive end [20, 23, 24, 26]. Consistent with their critical function, the sequences surrounding the PCSs are highly conserved among major HIV subtypes [27]. Drugs targeting Gag that impair protease-mediated processing at specific Gag cleavage sites have been developed [28]. A HIV vaccine targeting the viral protease cleavage sites (PCSs) has been proposed for its ability to generate antiviral immune responses, disrupt HIV maturation and limit target cell activation [10, 18, 27].

Simian immunodeficiency virus (SIV) infection of nonhuman primates (NHPs) is currently the best animal model to test HIV vaccine strategies or study HIV pathogenesis [29–42]. To evaluate a candidate HIV vaccine targeting the PCSs (PCS vaccine), we use female Mauritian cynomolgus macaques (MCMs) and SIVmac as a model. The PCS vaccine [35] consists of twelve 20-mer peptides overlapping the twelve PCSs of SIVmac239 [43–45]. These peptides were delivered with recombinant vesicular stomatitis virus (rVSV) [46] and nanoparticles (NANO) [47–51]. In parallel, we also evaluate a vaccine targeting full-length Gag and Env (Gag/Env vaccine).

The majority of HIV infections worldwide are acquired through the mucosal routes during sexual contact [52]. Women are especially vulnerable through vaginal exposure to HIV in seminal fluids and constitute more than half of all infections globally [53]. Therefore, inducing a protective immune response at mucosal sites, including the female genital tract, is extremely important in HIV vaccine development [53–56]. In this study, we evaluated the PCS vaccine in comparison with the Gag/Env vaccine in generating mucosal antibody responses to different immunogens, which may impact on the outcome of viral challenge [57].

## Materials and methods

### Ethics statement

Female Mauritian cynomolgus macaques (MCMs) were pair-housed within the same experimental group during the immunization phase of the study with visual and auditory access to other conspecifics. Paired monkeys lived in two adjacent standard stainless-steel primate cages (27”L x 27”W x 32”H per cage). Rooms were maintained at 65–75°F, 30–70% humidity, and on a 12:12 light-dark cycle (ON: 0600, OFF: 1800). Standard nonhuman primate chow with fruit or vegetables was provided daily. In addition, we provided foraging activities and physical environmental enrichment at least weekly for both activities. All animals were observed at least twice daily for health or welfare issues. Sedation (ketamine alone, or ketamine/dexmedetomidine, atipamezole for reversal) was provided during the experimental procedures. The experiments were approved by the University of Wisconsin IACUC protocol (G005765) in accordance with the US Animal Welfare Act and following the recommendations of the National Research Council *Guide for the Care and Use of Laboratory Animals*, *8th Edition* and the Weatherall report, *The Use of Nonhuman Primates in Research*. The Wisconsin National Primate Research Center is fully accredited by AAALAC under the University of Wisconsin, Division of Vice-Chancellor for Research and Graduate Education.

### Production of high titer rVSVpcs

The sequence of Simian immunodeficiency virus strain SIVmac239 was retrieved from the Los Alamos National Laboratory HIV database (http://www.hiv.lanl.gov). The nucleotide sequence encoding 20 amino acids (10 amino acids flanking each side of the cleavage site) overlapping each of the 12 PCSs of SIVmac239 was previously cloned in a recombinant vesicular stomatitis virus (rVSV) vector, pATX VSV-G, and packaged into rVSVpcs virus [35]. To generate large viral stocks for macaque immunization, VeroE6 cells were grown to 90% confluence in a T175 flask with 25 ml media, Dulbecco’s modified Eagle’s medium (DMEM) containing 10% fetal bovine serum (FBS), penicillin (100 U/ml), streptomycin (100 μg/ml) and L glutamine (2mM) (Invitrogen, CA, USA). 20 ml of the old culture media was removed and replaced with the same volume of fresh media 30 min before viral inoculation. Approximately 1×10^6^ plaque forming units (pfu) of rVSVpcs virus was then added to the cell culture and allowed to proliferate for 12–24 hours until 90–100% cell death was observed. The culture supernatants were harvested and centrifuged at 180 g/min for 5 min at room temperature to remove cellular debris.

To concentrate and purify rVSVpcs using density gradient ultracentrifugation, 20 ml of the supernatants were gently layered on top of 8 ml of equilibration buffer (20 mM Tris-HCl, 0.1 M NaCl and 0.1 mM EDTA) containing 20% sucrose (Fisher Scientific) in an Ultra-Clear™ centrifugation tube (Beckman Coulter). To make the equilibration buffer, 20 ml of Ultra Pure 1M Tris-HCl pH7.5 (Invitrogen), 20 ml of 5M NaCl (Invitrogen) and 200 μl of 0.5M EDTA pH8.0 (Ambion) were diluted with sterile water to 1L. Sucrose was freshly added to the equilibration buffer on the day of viral purification. Ultracentrifugation was performed in a Beckman Coulter XPN-80 Ultracentrifuge using the SW 32 Ti rotor at 27,000 rpm for 2h at 4°C. After carefully removing the supernatants, viral pellets were resuspended in 1 ml DMEM containing 10% FBS, and finally stored as 100 μl aliquots in a -80°C freezer.

Viral titers were calculated using the TCID_50_ calculator by Marco Binder (https://www.researchgate.net/file.PostFileLoader.html?id=58dad730f7b67ea37125593f&assetKey=AS%3A476999471898624%401490736944531), based on cytopathic effect of serially diluted viral stocks on VeroE6 cells.

### Packaging of PCS peptides into nanoparticles (NANOpcs)

Twelve synthetic 20mer peptides overlapping the PCSs of SIVmac239 were associated to a biodegradable nanoparticle system formed by chitosan and dextran sulfate, as previously described [35].

### Generation of rVSVGag/Env vaccine

Full-length Gag and Env coding sequences of SIVmac239 were synthesized and cloned in a Blue Heron pUC(-)MCS plasmid (BlueHeron Biotechnology, Bothell, WA, USA). Each gene sequence was flanked by an upstream MluI restriction site (AAACGCGT), Kozak sequence (GCCACC), start codon, and downstream stop codon and AvrII restriction site (CCTAGGTT). Using these restriction sites, the Gag and Env coding fragments were each sub-cloned into the rVSV vector pATX VSV-G, followed by confirmation with sequencing, and packaged into rVSVgag/env viruses, based on the previously described methods for rVSVpcs [35]. Large stocks of high-titer purified rVSVgag/env viruses were produced with the same methods as described above for rVSVpcs. As a control vaccine vector, wild type virus (rVSV) was similarly produced. To test SIV protein expression, supernatants from VeroE6 cell cultures infected with these rVSVs were analyzed by Western blot.

### Generation of NANOgag/env DNA vaccine

Using the above-mentioned Blue Heron pUC(-)MCS-Gag/Env plasmids as templates, full-length Gag and Env genes were PCR amplified with primers introducing an EcoRI restriction site upstream of the Kozak sequence and start codon and an XhoI restriction site downstream of the stop codon. These primers were:

Forward Gag primer: 5’CCGGAATTCGCCACCATGGGCGTGAGAAACTCCG3’

Reverse Gag primer: 5’CCGCTCGAGCTACTGGTCTCCTCCAAAGAGAG3’

Forward Env primer: 5’CCGGAATTCGCCACCATGGGATGTCTTGGGAATC-3’

Reverse Env primer: 5’CCGCTCGAGTCACAAGAGAGTGAGCTCAAGC-3’

The PCR products were then sub-cloned into a DNA vaccine vector, pVAX1, between the EcoRI and XhoI sites, followed by confirmation with sequencing.

The resulting full-length Gag and Env-coding DNA constructs, pVAX1-Gag and pVAX1-Env, were each packaged into DNA vaccine nanoparticles (NANOgag and NANOenv, also collectively named NANOgag/env when administered together), according to the ionotropic gelation technique previously published [58]. Chitosan (Heppe Medical Chitosan GmbH, Halle, Germany) and tripolyphosphate (TPP, Sigma-Aldrich, St. Louis, MO, USA) were separately dissolved in ultrapure water at a concentration of 0.625 mg/mL and 2 mg/mL, respectively. Then 0.7 mL of TPP at the concentration of 2 mg/mL was mixed with 2.1 mL of 0.33 mg/mL solution of plasmid. This mixture was slowly added over 11.2 mL of chitosan solution at a concentration of 0.625 mg/mL, under magnetic stirring. Nanoparticles were instantly formed upon the addition, and the mixture was kept under stirring for 10 minutes. For the freeze-drying process, 0.65 mL of a filtered solution of trehalose at 150 mg/mL was added to 6.5 mL of the nanoparticle suspension. Samples were frozen at -80°C and subsequently freeze-dried (Genesis 25 ES, VirTis Model-Wizard 2.0, SP Industries, USA). Prior to animal administration, the freeze-dried particles were resuspended by adding 0.65 mL of water, vortexing for 10 seconds and shaking horizontally for 10 minutes. The physical characteristics of the freeze-dried particles after resuspension included: size (NANOgag = 235 ± 4 nm and NANOenv = 225 ± 18 nm), polydispersity index (both ≈ 0.2) and zeta potential (NANOgag = +38 ± 3 mV and NANOenv = +39 ± 4 mV).

To test SIV protein expression, the Gag and Env DNA vaccine constructs were each used to transfect HEK293T cells using lipofectamine 2000 (Thermo Fisher Scientific, Waltham, MA, USA). HEK293 T cells were cultured to 90% confluence (in the same media as used for VeroE6 described above) in 6-well plates. Before transfection, the media were replaced with 2 ml of antibiotic-free DMEM containing 10% FBS. 2.5 μg DNA and 10 μl lipofectamine 2000 were diluted separately in 250 μl Opti-MEM (Thermo Fisher Scientific). The diluted DNA was mixed with the diluted lipofectamine 2000 and incubated for 5 min at room temperature. The DNA-lipid complex was then added to the cells. Transfected cells were harvested 24 h post transfection. Approximately 1×10^6^ cells were lysed with 50 μl RIPA lysis and extraction buffer (Thermo Fisher Scientific), then passed through a QIAshredder column (Qiagen) by centrifugation at 15,000 g for 2 min. The processed lysates were finally analyzed by Western blot.

### Vaccination

Three groups of eight female MCMs were used in the study. Group 1, the PCS vaccine group received rVSVpcs (viruses expressing PCS peptides) and NANOpcs (nanoparticles containing PCS peptides), Group 2, the Gag/Env vaccine group received rVSVgag/env (viruses expressing full-length Gag and Env proteins) and NANOgag/env (nanoparticles containing plasmid DNA encoding full-length Gag and Env), and Group 3, the Control group received vaccine vector controls (empty rVSV virus, and sterile water—the nanoparticle vehicle). One animal from the Gag/Env vaccine group was euthanized early due to severe health issues unrelated to vaccination, leaving seven animals in this group to complete the study. The vaccination procedure consisted of a prime with rVSVs at week 0, the first boost with rVSVs + NANOs at week 6, the second boost with NANOs at week 16, the third boost with rVSVs + NANOs at week 51 and the fourth boost with rVSVs at week 72, respectively. All rVSVs were administered intramuscularly via the quadriceps muscle, alternating L and R for successive vaccinations. The dose was at 1×10^6^ pfu of each rVSVpcs per animal of the PCS Vaccine group or 6×10^6^ pfu of rVSVgag and 6×10^6^ pfu of rVSVenv per animal of the Gag/Env Vaccine group, except that for the 4^th^ boost 1×10^8^ pfu/rVSV type/animal were administered. All NANOs were administered intranasally, with each animal receiving NANO-delivered 50 μg peptide for each of the twelve PCS peptides (the PCS vaccine group) or NANO-delivered plasmid DNA encoding Gag (500 μg) and Env(500 μg) (the Gag/Env vaccine group).

### Cervicovaginal lavage (CVL) sample collection

The vaginal lumen of sedated animals was rinsed with 2–6 ml of phosphate buffered saline (PBS) non-traumatically using a needleless syringe. Within this volume range, the total amount of PBS used varied from animal to animal because of the varying sizes of their cervical vaults. The PBS was administered until the vault is full and then collected back into the same syringe. The fluid was gently flushed five times using the same syringe and repeated until 2–4 ml of fluid was collected. To take the volume variation into account, mucosal antibody concentrations were all normalized to total protein concentrations of the collected samples. The CVL sample collection was performed non-traumatically with extreme caution to avoid blood contamination from tissue damage. In addition, the animals were examined every day for menses and we have kept a daily menses record during the study. Due to variable dates of menstruation among individual female macaques and fixed dates of sampling schedule, it was practically difficult to avoid menstruation dates in all CVL collections. Indeed a small percentage of samples (11.25%) were collected on menstruation dates. To rule out effect of any potential menses blood contamination, we excluded data of the samples collected during menstruation from final analysis. All CVL sample aliquots were stored at -80°C until use.

### Bio-Plex antibody assay

CVL IgG antibodies to SIV antigens were quantified by following a previously published Bio-Plex multiplexed protocol [35]. CVL total protein concentrations were determined using a NanoOrange® protein quantitation kit (Thermo Fisher Scientific) according to the manufacturer’s protocol. The IgG antibody level was normalized with the total protein concentration.

### Western blot

Western blot was used to detect: (1) SIV Gag and Env protein antigens expressed from rVSV and DNA Gag/Env vaccines; and (2) Vaccination-induced antibodies to purified recombinant Gag and Env proteins (NIH AIDS reagent program). Western blot was conducted following a previously published method [35] with slight modifications. For detection of antigens, 26 μl of supernatants from rVSV-infected VeroE6 cell culture, or lysates of DNA vaccine-transfected HEK293T cells equivalent to 5×10^5^ cells, were diluted and denatured in the format of a 40 μl NuPAGE sample and loaded for SDS-PAGE, followed by blotting. Gag and Env protein antigens were probed with standard mouse monoclonal antibodies (NIH AIDS Reagent Program) as listed in the published method [35]. For detection of antibodies, 1 μg of purified recombinant SIV proteins (NIH AIDS Reagent Program), SIVmac251 Gag (Catalog 1845) and SIVmac239 Env (Catalog 12797), were used as standard antigens for blotting. CVL samples from the control, PCS vaccine and Gag/Env vaccine groups of animals were 1:10 diluted and used as primary antibodies. IgG antibodies bound to the antigens were then detected using an anti-monkey IgG-HRP secondary antibody [35].

### Statistical analysis

Mann Whitney's test was conducted using GraphPad Prism 7.04 to compare antibody responses among the PCS vaccine, Gag/Env vaccine and control groups. A *p* value less than 0.05 was defined as significant.

## Results

### Generation of the PCS vaccine and the Gag/Env vaccine

**Fig 1:**
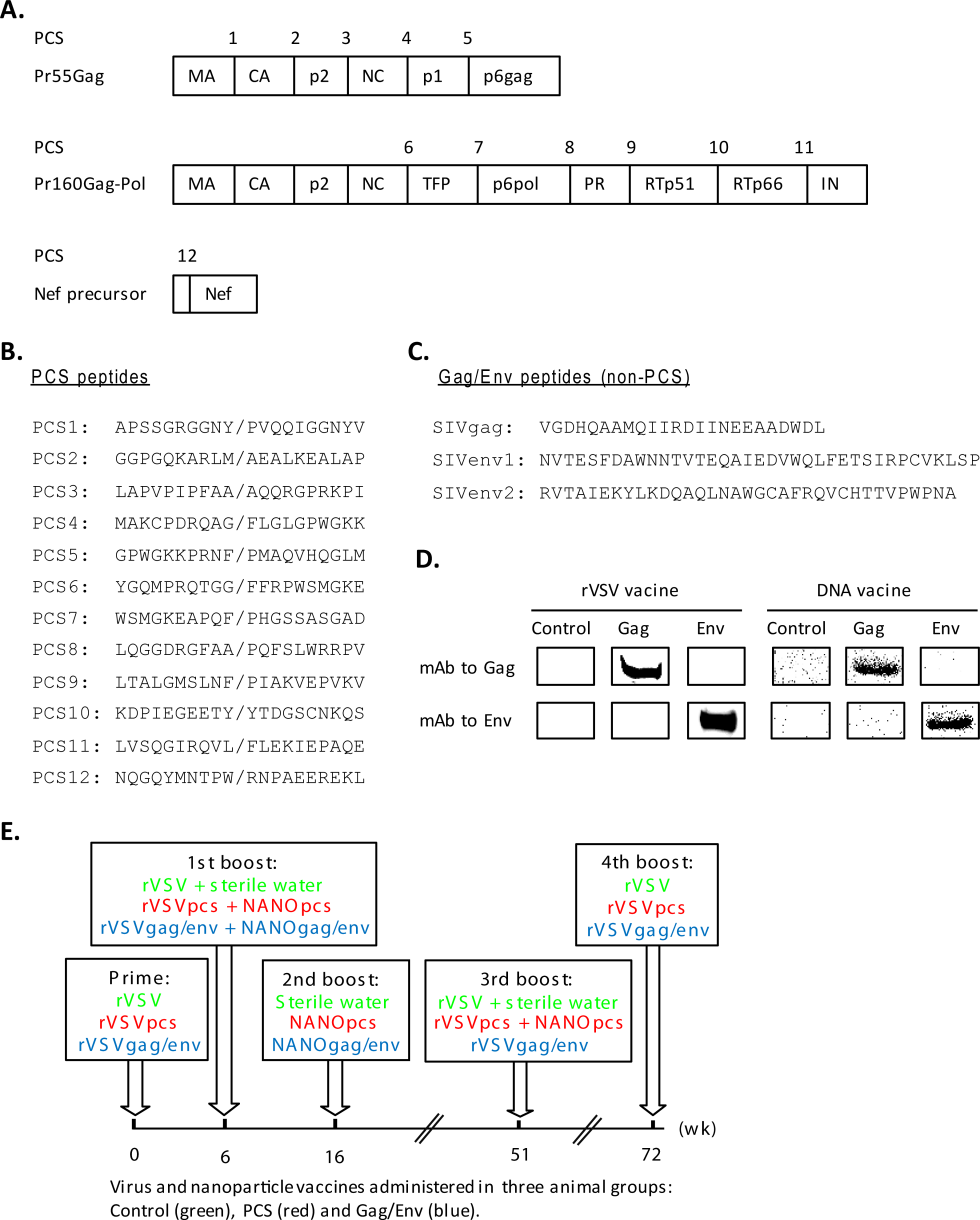
Vaccines targeting SIV protease cleavage sites (PCSs) or full Gag and Env proteins. (A) Diagram of the twelve protease cleavage sites (PCS1 through PCS12), located on three SIV polyproteins (Pr55Gag, Pr160Gag-Pol and Nef precursor), not drawn to scale. MA: matrix; CA: capsid; NC: nucleocapsid; TFP: transframe protein; PR: protease; RT: reverse transcriptase; and IN: integrase. (B) Peptide sequences of SIV immunogens in a conserved element vaccine targeting the PCSs (the PCS vaccine). Each sequence corresponds to -10 through +10 amino acid positions flanking each cleavage site. Slash (/) indicates the site of protease cleavage. These sequences were confirmed to be specific for SIV by NCBI protein BLAST and conserved among multiple SIV strains. The peptide immunogens were delivered as recombinant vesicular stomatitis viruses (rVSVpcs) and nanoparticles (NANOpcs). Peptide antigens with these sequences were also used in a Bio-Plex multiplexed assay to detect anti-PCS antibodies. (C) Sequences of three Gag or Env (non-PCS) peptides used in Bio-Plex to detect anti-Gag or Env antibodies, including one Gag peptide, named SIVgag, and two Env peptides, named SIVenv1 and SIVenv2. (D) Western blot analyses of protein expression from a full-length Gag and Env-based vaccine (the Gag/Env vaccine). VeroE6 cells were infected with recombinant vesicular stomatitis viruses (rVSVs) carrying full Gag or Env gene of SIVmac239 (rVSVgag/env) and the culture supernatants were analyzed by Western blot to detect Gag or Env protein expression using standard monoclonal antibodies (mAb, NIH AIDS Reagent Program) to Gag or Env. The full Gag and Env genes were also cloned into pVAX1 (a DNA vaccine vector), respectively, followed by NANO packaging (NANOgag/env). HEK293T cells were transfected with these DNA vaccines and analyzed by Western blot. (E) Vaccination scheme. Three groups (Control, PCS vaccine and Gag/Env vaccine) of eight female MCMs per group were primed and boosted on indicated weeks (wk). The Control group received empty rVSV virus and sterile water. One animal from the Gag/Env vaccine group was euthanized early due to severe health issues unrelated to vaccination, leaving seven animals in this group to complete the study. rVSV control vector (rVSV), rVSVpcs or rVSVgag/env was administered intramuscularly. NANO control vector (sterile water), NANOpcs or NANOgag/env was administered intranasally.

The PCS vaccine consists of twelve 20-amino acid peptides each overlapping one of the twelve PCSs of SIVmac239 (PCS1 through PCS12) **(Fig 1A and 1B)**. The PCS immunogens were delivered in recombinant vesicular stomatitis virus (rVSVpcs) and nanoparticles (NANOpcs). rVSV is a non-pathogenic, replication competent viral vector. It can induce robust humoral and cellular immune responses, and unlike adenoviral vectors, lacks pre-existing human immunity [46]. The success of the vector was demonstrated by the safety and protective efficacy of an Ebola virus vaccine [59, 60]. Biodegradable NANO materials have great capacity for mucosal vaccination [47, 48, 50, 51, 61] as demonstrated by its successful nasal delivery of tetanus toxoid [61, 62] and hepatitis B surface antigen [47, 49, 63] subunit vaccines. For simplicity, the rVSVpcs and NANOpcs were collectively referred to as the PCS vaccine, as both were used in combination to immunize the same group of animals. The PCS vaccine was shown to induce plasma antibodies to the PCS peptides in a pilot study [35]. For comparison, we generated a rVSV vaccine with full-length Gag and Env genes of SIVmac239 (rVSVgag/env). The expression of Gag and Env proteins by rVSVgag/env was confirmed by Western blot using VeroE6 cell cultures infected by these viruses **(Fig 1D)**. Gag and Env-expressing DNA vaccines in pVAX1 were also generated to be delivered with nanomaterial for boosting immune response to Gag and Env (NANOgag/env). The expression of Gag and Env proteins from the DNA vaccine constructs was confirmed by Western blot in transfected HEK293T cells **(Fig 1D)**. The rVSVgag/env and NANOgag/env in combination were named the Gag/Env vaccine, to be compared with the PCS vaccine.

### The immunization scheme

We carefully considered the routes of vaccination as they are well known factors to impact on the localized induction of immune responses. In general, systemic delivery of immunogens tends to elicit systemic responses and mucosal delivery of immunogens tends to induce mucosal immune responses [53]. However, systemically delivered viral vectors can also induce mucosal immune responses to HIV or SIV [64, 65]. Mucosal vaccination at one site stimulates immune responses in all mucosal sites, as well as systemic immune responses [53]. Intranasal immunization was reported to be the most effective at eliciting immune responses in the female genital tract [66], and this route induces greater IgG systemic responses than other mucosal routes, oral, rectal or vaginal [67]. It was proposed that systemic prime followed by mucosal boosting may help prevent induction of mucosal tolerance by initial mucosal vaccination, and should elicit both systemic and mucosal antibodies [53]. Taking all these into considerations, we chose to use rVSVs for systemic prime and boost through the intramuscular route (i.m.) and NANOs for mucosal boost through the intranasal route (i.n.). The resulting vaccination scheme consisted of a prime with rVSVs and four boosts with combinations of rVSVs and NANOs, as illustrated in **Fig 1E**.

### Mucosal SIV-specific IgG antibodies elicited by vaccine modalities targeting different immunogens of SIVmac239

Protective mucosal immune responses to HIV is critical in preventing its mucosal transmission [52, 53]. In several passive and active NHP immunization experiments mucosal IgG antibodies showed protective effect against simian-human immunodeficiency virus (SHIV) acquisition (57–65). Therefore, we analyzed mucosal antigen-specific IgG responses after MCMs were primed and boosted with the PCS vaccine or the Gag/Env vaccine following the vaccination scheme illustrated in **Fig 1E**. Cervicovaginal lavage (CVL) samples were collected and measured for IgG responses to the twelve PCS peptides and three Gag/Env (non-PCS) peptides, SIVgag, SIVenv1 and SIVenv2 (**Fig 1C**) by Bio-Plex and to Gag and Env proteins by Western blot.

**Fig 2:**
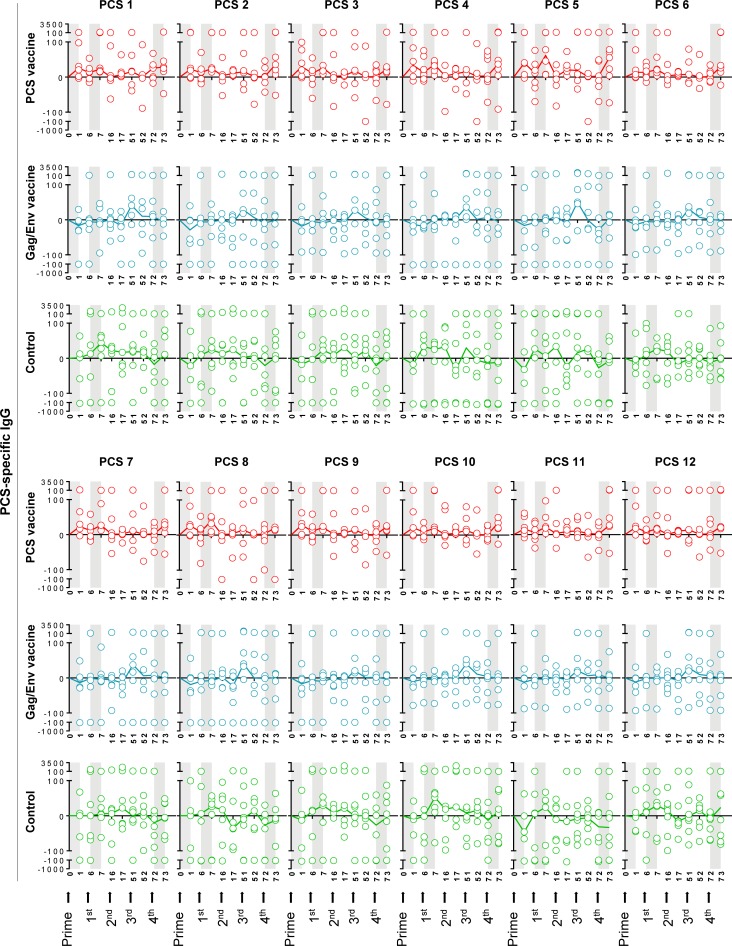
Dynamics of PCS-specific IgG antibodies in cervicovaginal secretions. Cervicovaginal lavage (CVL) samples from the vaccination experiments illustrated in Fig 1E were quantified for levels of IgG antibodies to each PCS peptide (y axis, expressed as ratios of anti-PCS IgG concentration to total protein concentration ×10^9^) by a Bio-Plex multiplexed antibody assay, at indicated time points (x axis, weeks post prime). The Control group received empty rVSV virus and sterile water. Data are shown as each value from individual animals with median line, following subtraction of pre-vaccination values. Total animal numbers per group examined are n = 8 (Control or PCS group) or n = 7 (Gag/Env group). However, for technical stringency, animal samples collected on menstruation dates were excluded from analysis to rule out any potential menses blood contamination. As a result, for some of the data points in the graph, fewer individual values than the total animal number (n = 8 or n = 7) were available. Grey areas indicate a week interval post prime, 1^st^ boost or 4^th^ boost, with trends of antibody increase in the PCS vaccine group in response to vaccination. The trends did not reach statistical significance.

**Fig 3:**
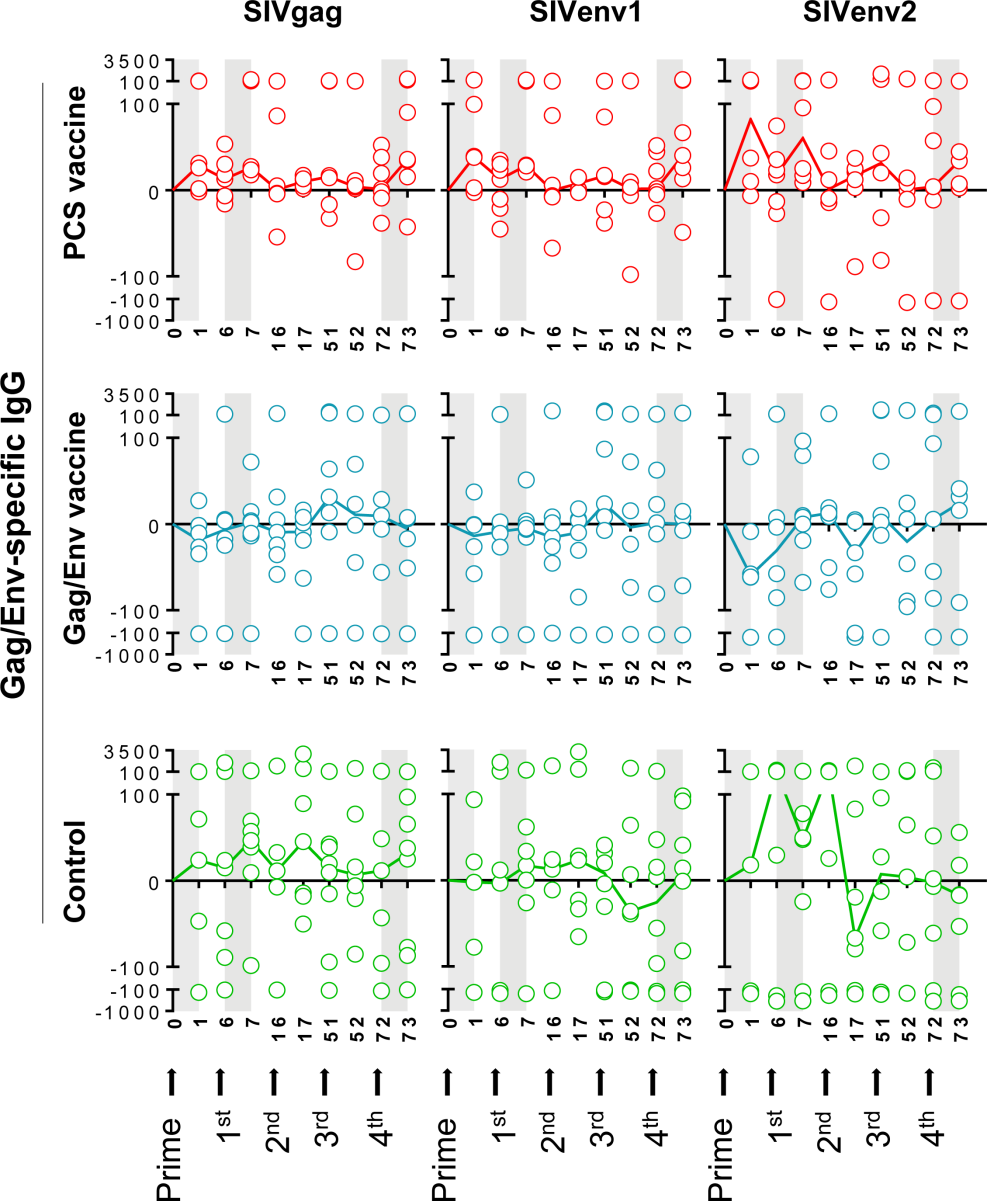
Dynamics of Gag/Env-specific IgG antibodies in cervicovaginal secretions. Cervicovaginal lavage (CVL) samples from the vaccination experiments illustrated in Fig 1E were quantified for IgG antibodies to Gag/Env (non-PCS) peptides, SIVgag, SIVenv1 and SIVenv2 (y axis, expressed as ratios of anti-non-PCS IgG concentration to total protein concentration ×10^9^) by a Bio-Plex multiplexed antibody assay, at indicated time points (x axis, weeks post prime). The Control group received empty rVSV virus and sterile water. Data are shown as each value from individual animals with median line, following subtraction of pre-vaccination values. Total animal numbers per group examined are n = 8 (Control or PCS group) or n = 7 (Gag/Env group). However, for technical stringency, animal samples collected on menstruation dates were excluded from analysis to rule out any potential menses blood contamination. As a result, for some of the data points in the graph, fewer individual values than the total animal number (n = 8 or n = 7) were available. Grey areas indicate a week interval post prime, 1^st^ boost or 4^th^ boost, with trends of antibody increase in the PCS vaccine group in response to vaccination. The trends did not reach statistical significance.

We first analyzed the dynamics of antibodies to each of the twelve PCS peptides (PCS1 –PCS12) throughout the vaccination procedure. These antibodies showed trend of increase in the PCS vaccine group compared to the Gag/Env vaccine and Control group after prime, the 1^st^ boost and the 4^th^ boost (**Fig 2**). Similar patterns were observed in the dynamics of antibodies to non-PCS Gag/Env peptides (SIVgag, SIVenv1 and SIVenv2) (**Fig 3**). We also observed variable antibody responses to PCS peptides among individual animals in the PCS vaccine (**Figure A in S1 File**), Gag/Env vaccine (**Figure B in S1 File**) and Control (**Figure C in S1 File**) groups, by comparing antibody levels between the baseline and one week after the last boost (4th). Six out of seven animals (85.7%) from the PCS vaccine group **(Figure A in S1 File)** and three out of six animals (50%) from the Gag/Env vaccine group **(Figure B in S1 File)** showed consistent increase in IgG antibodies to all PCS peptides. In contrast, only one out of eight animals (12.5%) from the control group showed a similar pattern **(Figure C in S1 File)**. Similar results were also seen in antibody responses to Gag/Env peptides (**Figures D-F in S1 File**).

**Fig 4:**
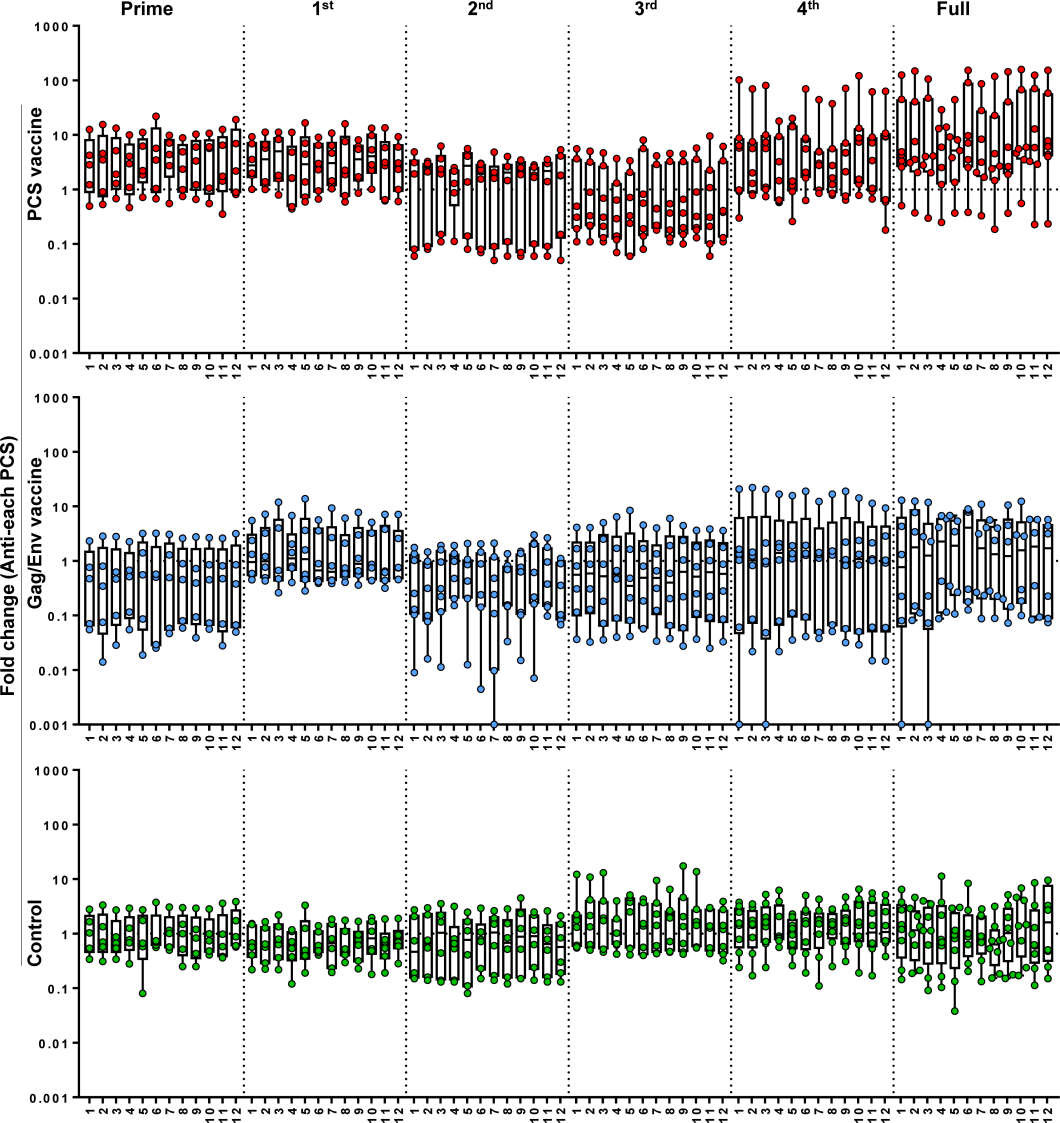
Fold changes of mucosal antibodies to each PCS peptide in response to vaccinations. Graphs show fold changes of mucosal IgG antibodies to each PCS peptide (1 through 12) between the time of a prime/boost and one week after that single prime/boost (Prime, 1^st^, 2^nd^, 3^rd^ or 4^th^), or between the baseline (the start of the full vaccination procedure) and one week after the last boost (the end of the full vaccination procedure) (Full). The Control group received empty rVSV virus and sterile water. Data are shown as each value from individual animals with interquartile range and median line. Total animal numbers per group examined are n = 8 (Control or PCS group) or n = 7 (Gag/Env group). However, for technical stringency, animal samples collected on menstruation dates were excluded from analysis to rule out any potential menses blood contamination. As a result, for some of the data points in the graph, fewer individual values than the total animal number (n = 8 or n = 7) were available. Fold changes of antibodies in response to prime, the 1^st^ boost, the 4^th^ boost and the full vaccination procedure showed trends of increase in the PCS vaccine group, compared to the other groups. The trends did not reach statistical significance.

We quantified the effect of prime, each boost and the full vaccination process on antibody responses to each PCS. The PCS vaccine group showed trends of higher fold increase of antibodies (after prime, the 1^st^ boost, the 4^th^ boost, or the full vaccination process) than the other groups (**Fig 4**).

**Fig 5:**
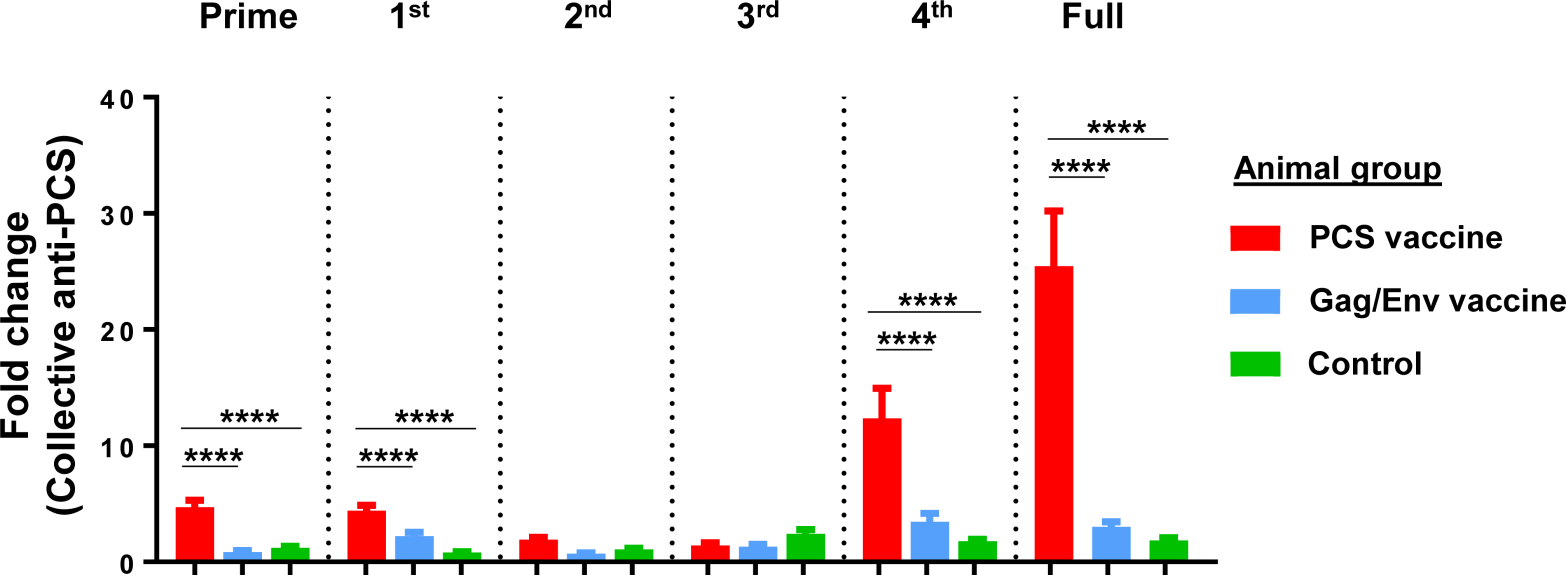
Fold changes of collective anti-PCS antibodies in response to vaccinations. Mucosal IgG antibodies to all individual PCS types (PCS1 through PCS12) were collectively treated as anti-PCS antibodies for calculation. Graph shows fold changes between the time of a prime/boost and one week after that single prime/boost (Prime, 1^st^, 2^nd^, 3^rd^ or 4^th^), or between the baseline (the start of the full vaccination procedure) and one week after the last boost (the end of the full vaccination procedure) (Full). The Control group received empty rVSV virus and sterile water. Total animal numbers per group examined are n = 8 (Control or PCS group) or n = 7 (Gag/Env group). However, for technical stringency, animal samples collected on menstruation dates were excluded from analysis to rule out any potential menses blood contamination. Bars represent mean ± SEM. The PCS vaccine group demonstrated significantly higher fold induction of anti-PCS antibodies than the Gag/Env vaccine and Control groups, in response to prime, the 1st boost, the 4th boost and the full vaccination procedure, as determined by Mann Whitney's test: **** p < 0.0001.

We then calculated the effect of vaccines on total antibody responses to all PCSs by including antibodies to all PCS sites collectively as anti-PCS antibodies since they all target the viral protease cleavage function. The fold changes of total anti-PCS antibodies, in response to prime, the 1^st^ boost, the 4^th^ boost or the full vaccination process, were significantly higher in the PCS vaccine group than those in the Gag/Env vaccine group and the Control group (p < 0.0001) (**Fig 5**). Among these, the mean fold changes induced by the 4^th^ boost were 12.353 (PCS vaccine), 3.465 (Gag/Env vaccine) and 1.792 (Control), and those induced by the full vaccination process were 25.466 (PCS vaccine), 3.005 (Gag/Env vaccine) and 1.853 (Control) (**Fig 5**). These results indicated that the PCS vaccine can effectively induce mucosal anti-PCS antibodies at the female genital tract and has a stronger effect than the Gag/Env vaccine.

**Fig 6:**
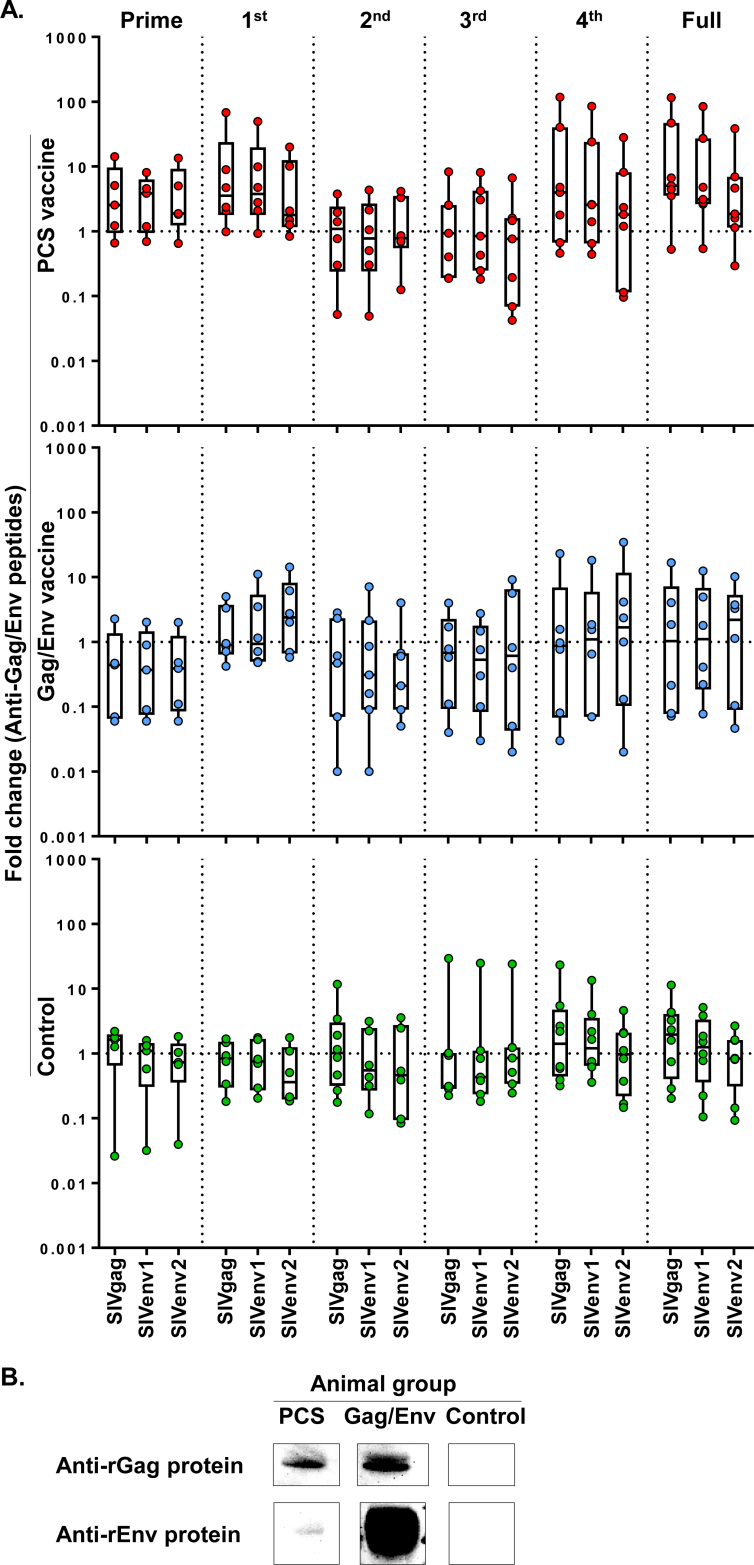
Induction of antibodies to Gag/Env antigens. (A) Graphs show fold changes of mucosal IgG antibodies to Gag/Env (non-PCS) peptides (SIVgag, SIVenv1 and SIVenv2) between the time of a prime/boost and one week after that single prime/boost (Prime, 1^st^, 2^nd^, 3^rd^ or 4^th^), or between the baseline (the start of the full vaccination procedure) and one week after the last boost (the end of the full vaccination procedure) (Full). The Control group received empty rVSV virus and sterile water. Data are shown as each value from individual animals with interquartile range and median line. Total animal numbers per group examined are n = 8 (Control or PCS group) or n = 7 (Gag/Env group). However, for technical stringency, animal samples collected on menstruation dates were excluded from analysis to rule out any potential menses blood contamination. As a result, for some of the data points in the graph, fewer individual values than the total animal number (n = 8 or n = 7) were available. Fold changes of antibodies to SIVgag and SIVenv1 in response to prime, the 1^st^ boost, the 4^th^ boost and the full vaccination procedure showed trends of increase in the PCS vaccine group, compared to the other groups. The trends did not reach statistical significance. (B) Reactivity of mucosal IgG antibodies to Gag and Env proteins. Western blot membranes containing purified recombinant Gag (rGag) or Env (rEnv) protein (NIH AIDS Reagent Program) were used to probe anti-Gag/Env IgG antibodies in CVL samples, collected at one week after the last boost from animals of the PCS vaccine (animal ID: cy0759), Gag/Env vaccine (animal ID: cy0784) and Control (animal ID: cy0779) groups, respectively.

We also examined the effect of vaccines on antibody responses to Gag/Env. The PCS vaccine group showed trends of higher fold increase in antibodies to two non-PCS Gag/Env peptides, SIVgag and SIVenv1, after prime, the 1^st^ boost, the 4^th^ boost, and the full vaccination process, in comparison to the Control group (**Fig 6A**). The fold increase of antibodies to the three non-PCS Gag/Env peptides was not apparent in the Gag/Env group or the Control group (**Fig 6A**). We then tested antibody reactivity by Western blot using Gag and Env proteins, which were expected to detect antibodies that recognize epitopes not limited to the three Gag/Env peptides. Antibodies of vaccinated animals from the PCS and Gag/Env vaccine groups recognized purified recombinant Gag protein (rGag) **(Fig 6B)**. While antibodies from the Gag/Env group showed strong reactivity to purified recombinant Env protein (rEnv), those from the PCS vaccine group demonstrated weak but clear reactivity to rEnv **(Fig 6B)**. These results indicated that the PCS and Gag/Env vaccines can induce mucosal IgG antibodies to Gag and Env.

### Cross-reactivity of the vaccine-induced antibodies

Bio-Plex antibody assays suggested that the PCS vaccine, which delivers PCS peptides, induced antibodies that recognize non-PCS Gag/Env peptides (**Figs 3 and 6A**). The antibody cross-reactivity was supported by Western blot analyses demonstrating that these antibodies reacted to rGag and rEnv proteins **(Fig 6B)**. The rGag protein contains peptide sequences of PCSs (PCS1 through PCS5) (**Fig 1**), thus it was expected that the antibodies induced by the PCS vaccine could recognize Gag. However, rEnv does not contain any PCS sequence **(Fig 1)**, therefore the induction of anti-Env antibodies by the PCS vaccine was unexpected. While the underlying mechanism(s) of the cross-reactivity remain to be understood, these data indicated that the PCS vaccine can generate antibodies to both PCS peptides and Env.

## Discussion

In this study we examined mucosal antibodies induced by two different modalities of candidate HIV/SIV vaccines, a vaccine targeting short peptide sequences overlapping the 12 protease cleavage sites and a vaccine targeting full Gag and Env of SIVmac239. Since 90% of HIV transmissions occur through the mucosal route [68] and male to female sexual transmissions account for more than half of all HIV infections [53], it is important to test whether a candidate vaccine can induce mucosal immune responses to HIV/SIV antigens. Our study showed that both the PCS vaccine and Gag/Env vaccine can induce cervicovaginal mucosal IgG antibodies to SIV antigens, including PCSs, Gag and Env. The PCS vaccine preferentially generated mucosal IgG antibodies to the PCS peptides, whereas the Gag/Env vaccine generated much stronger mucosal IgG antibodies to Env.

Most of current vaccine studies on anti-HIV antibodies are focused on Env-specific antibodies. However, it was also shown that antibodies to Gag and Pol correlated with natural and post vaccination HIV control [69–74], suggesting that antibodies targeting Gag and Pol could be protective. Not only Env, but also Gag and Pol were included in the vaccine used for the RV144 trial, the only vaccine so far with efficacy against HIV acquisition [2]. A previous preclinical study also showed that Gag was required for protection against SIVsmE660 challenge [75]. Potential protective effect of the mucosal antibodies to PCSs, Gag and Env induced by different modalities of vaccines in the current study will be evaluated in repeated low dose SIVmac251 intravaginal challenges.

We observed that without SIV-specific immunization, antibodies with reactivity to SIV antigens can be present in some MCMs (at variable levels). The mechanism(s) for generating these antibodies remain to be understood. We speculate that endogenous or environmental antigens may induce antibodies with cross-reactivity to SIV antigens and subject to regulation by external stimuli [35, 37]. Our results also showed that the PCS vaccine elicited mucosal antibodies not only to the PCSs but also to non-PCS Gag and Env antigens. While several possibilities may account for this observation [35, 37], one of them may be the cross-reactivity of the anti-PCS antibodies to the non-PCS antigens [76]. Although there is no similarity in primary sequences between the PCS and non-PCS peptides, they may have similar conformational structures that could contribute to cross-reactivity [76]. It is important to note that the induction of antibodies with reactivity to non-PCS peptides did not distract the PCS vaccine away from its intended targets, the PCSs, since the PCS vaccine was shown to effectively elicit antibodies to these targets. The cross-reactivity of the PCS vaccine-induced antibodies expands the antigen spectrum to additional Gag and Env epitopes. As a result, the PCS vaccine may have the potential to target both PCS-based viral maturation and Env-mediated viral entry. These will need to be further investigated.

In conclusion, our study showed that the PCS vaccine and Gag/Env vaccine can induce mucosal IgG responses to SIV immunogens, including PCS peptides and Gag and Env proteins. The effect of these vaccine-induced mucosal antibodies in protecting macaques from pathogenic SIVmac251 low dose intravaginal challenges will be determined in on-going studies.

## Supporting information

S1 File A single PDF file including Figures A through F. (PDF)
