# Supplementary material for: Mucosal antibody responses to vaccines targeting SIV protease cleavage sites or full-length Gag and Env proteins in Mauritian cynomolgus macaques
Source: PLoS One. 2018 Aug 28;13(8):e0202997. doi: 10.1371/journal.pone.0202997 (PMC6112674; doi:10.1371/journal.pone.0202997)
Supplement: S1 File — (PDF) [file pone.0202997.s001.pdf]

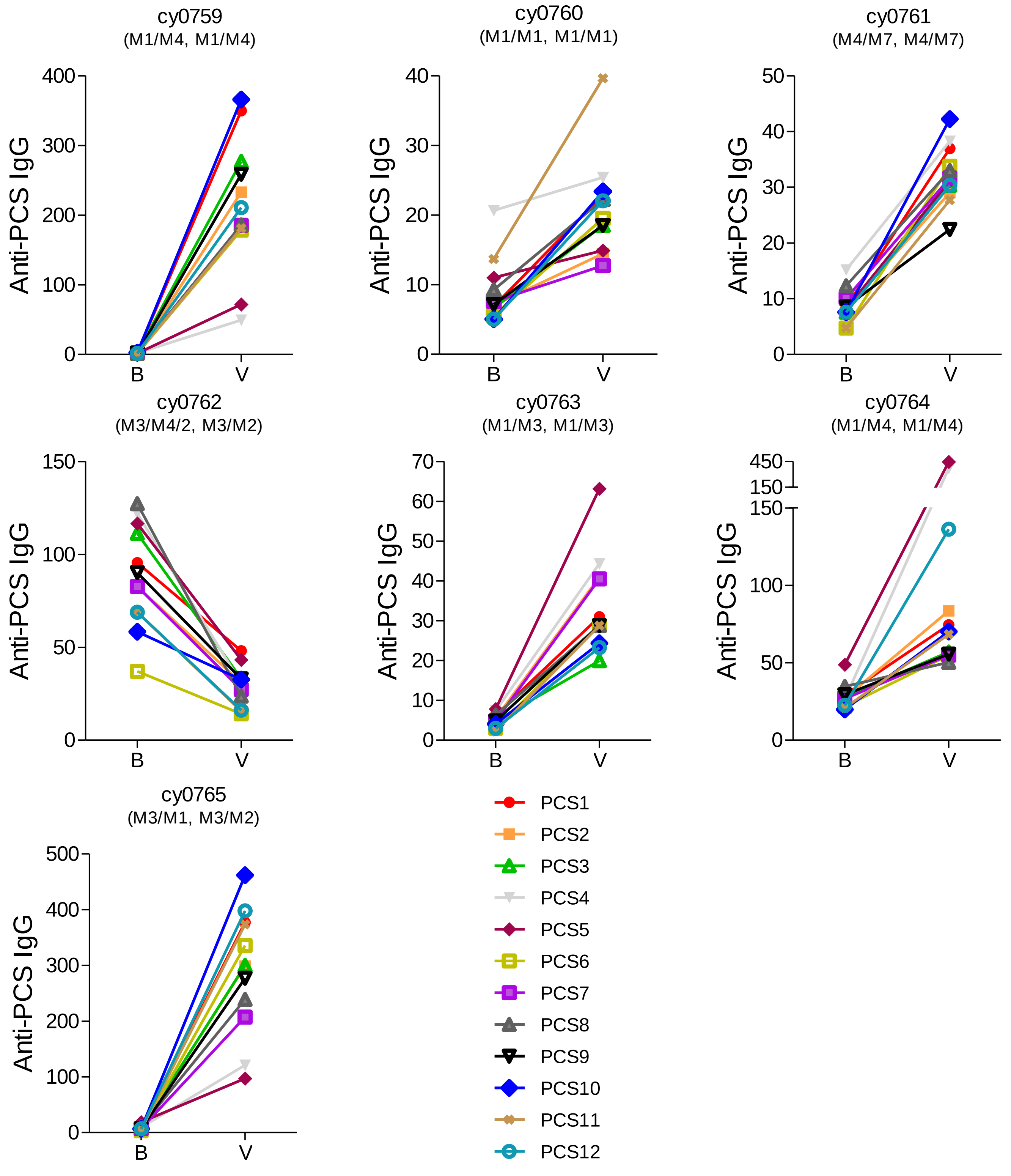

Figure A. The PCS vaccine group: Mucosal IgG responses to PCSs in each individual animal. Cervicovaginal lavage (CVL) samples of the PCS vaccine group animals from the vaccination experiments illustrated in Figure 1E were analyzed for antibodies to PCS peptides using a Bio-Plex multiplexed antibody assay. Each panel represents one animal, with animal ID and MHC haplotypes (MHC I, MHC II) shown on top. Data from one animal (ID cy0758) were excluded due to sample collection on concurrent menstruation dates. Antibody levels (ratios of anti-PCS IgG concentration to total protein concentration, timed by 10^9) in each animal were compared between the baseline (indicated as “B” on x axis) and after the vaccination procedure (one week after the last boost, indicated as “V” on x axis). Each line represents IgG antibody levels to one PCS type, connecting data points before and after vaccination.

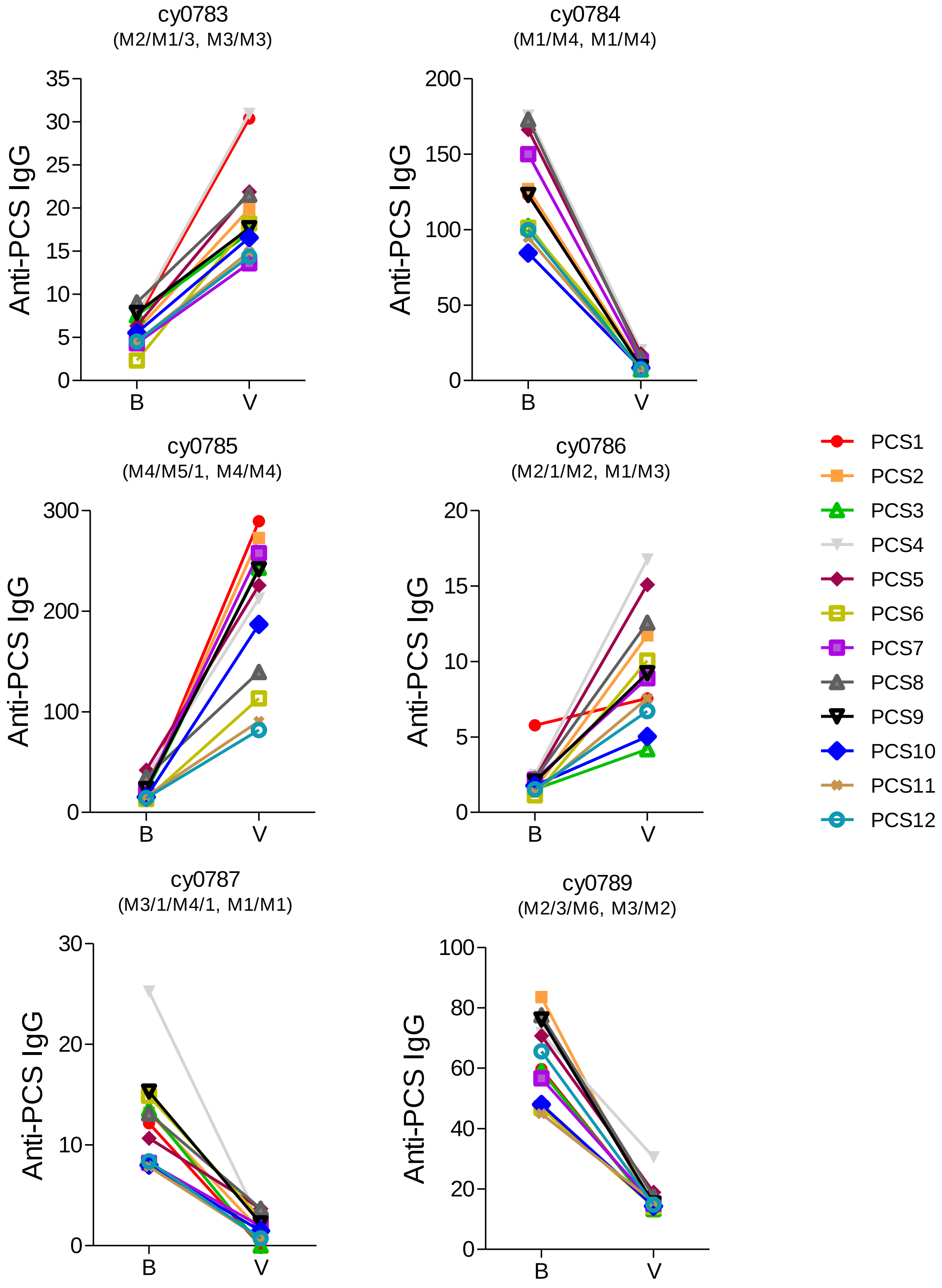

Figure B. The Gag/Env vaccine group: Mucosal IgG responses to PCSs in each individual animal. Antibodies to PCS peptides were analyzed and graphed as in Figure A. Data from two animals were excluded due to the following reasons: cy0782 was euthanized early due to health issues unrelated to vaccination. Sample collection of cy0788 involved concurrent menstruation dates.

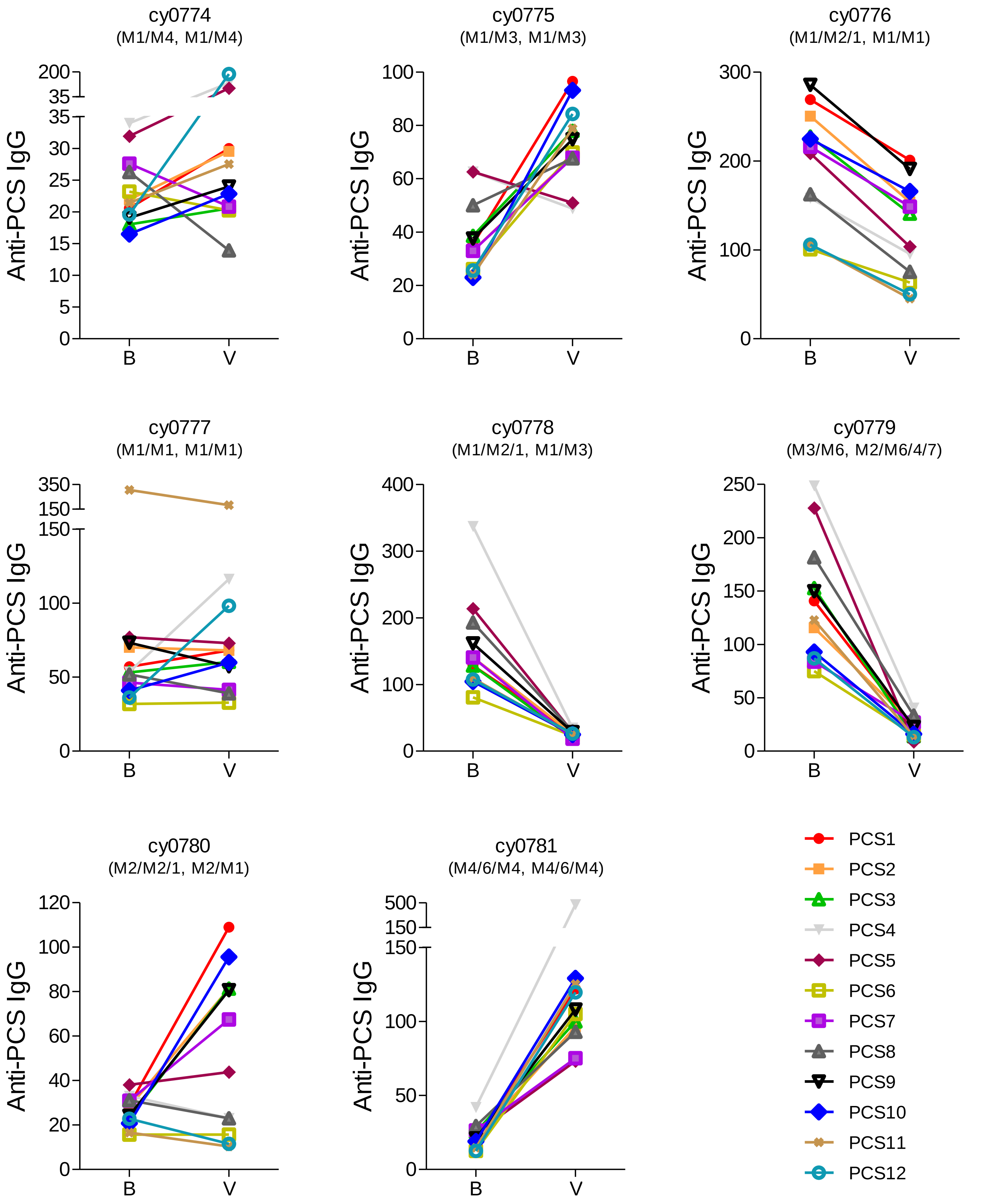

Figure C. The Control group: Mucosal IgG responses to PCSs in each individual animal. Antibodies to PCS peptides were analyzed and graphed as in Figure A.

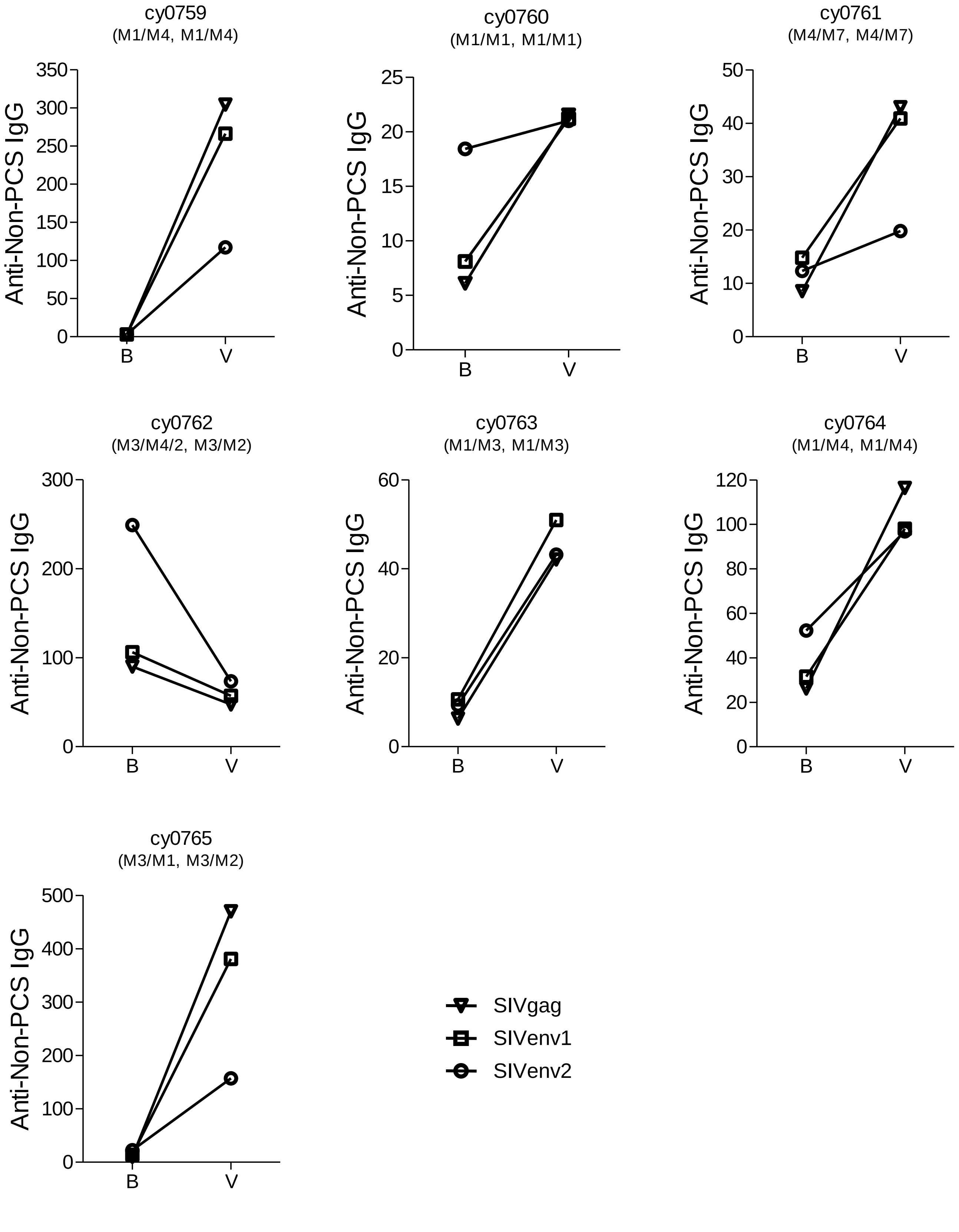

Figure D. PCS vaccine group: Mucosal IgG responses to Gag/Env peptides (non-PCS) in each individual animal. Antibodies to Gag/Env peptides were analyzed and graphed similarly to Figure A.

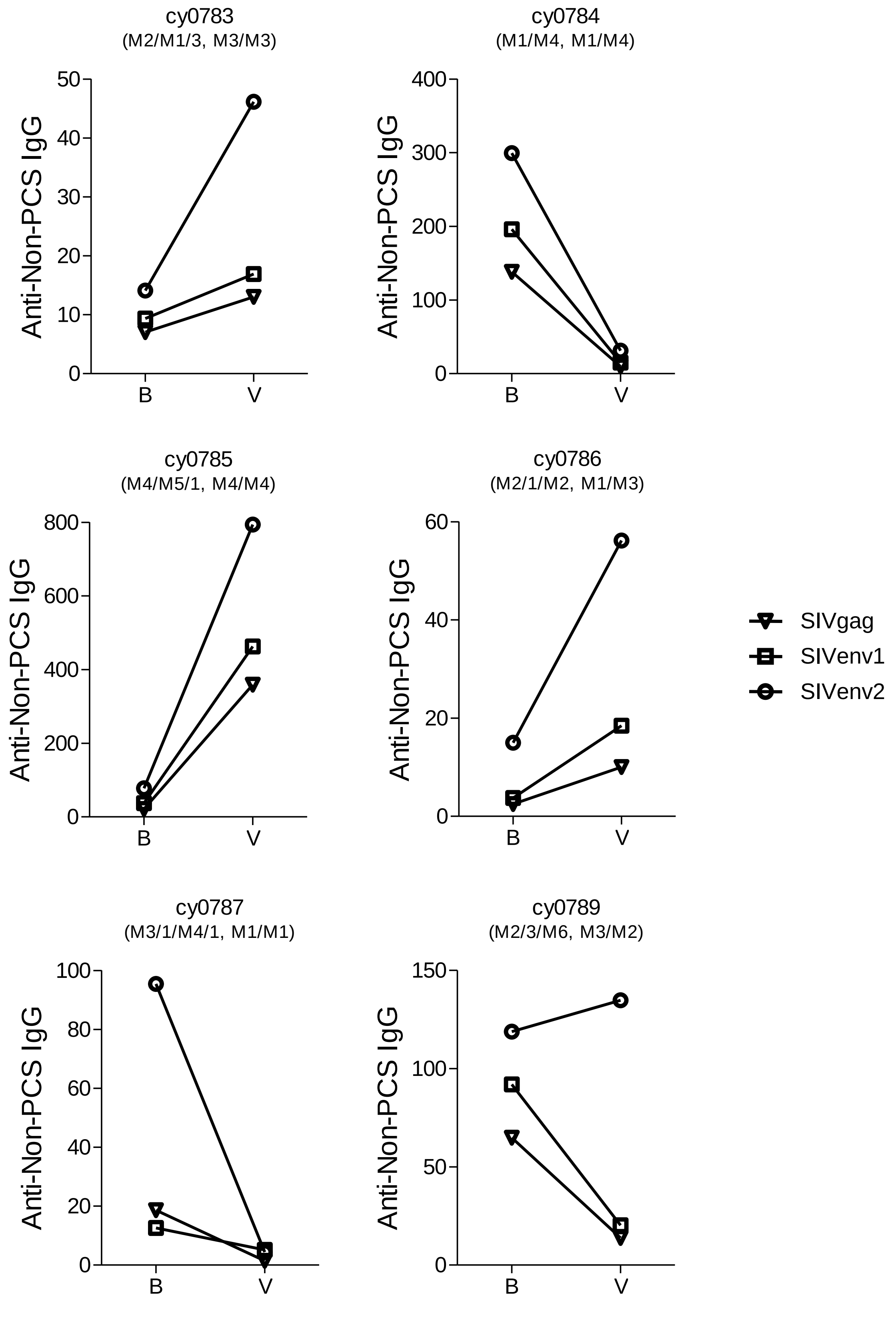

Figure E. Gag/Env vaccine group: Mucosal IgG responses to Gag/Env peptides (non-PCS) in each individual animal. Antibodies to Gag/Env peptides were analyzed and graphed similarly to Figure B.

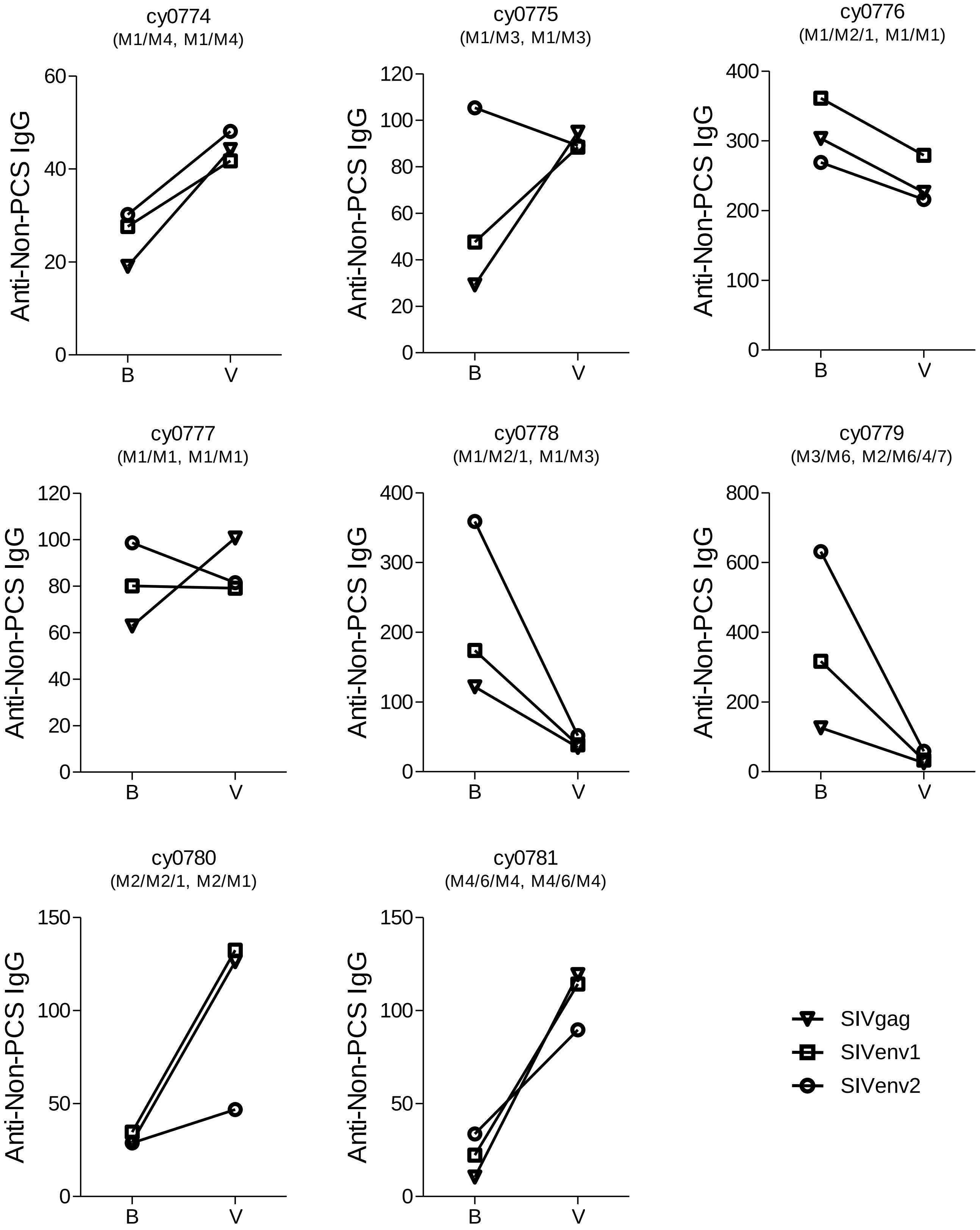

Figure F. Control group: Mucosal IgG responses to Gag/Env peptides (non-PCS) in each individual animal. Antibodies to Gag/Env peptides were analyzed and graphed similarly to Figure C.
